# Prevalence of autistic traits and their relationships with other psychopathological domains in young adults seeking psychiatric attention: a cluster analysis

**DOI:** 10.1192/j.eurpsy.2025.545

**Published:** 2025-08-26

**Authors:** G. Ingrosso, B. Demartini, F. Serio, V. Nisticò, G. Broglia, A. Bertani, R. Faggioli, O. Gambini, G. Massimetti, L. Dell’Osso, B. Carpita

**Affiliations:** 1 Department of Health Sciences, University of Milan; 2 University of Milan, “Aldo Ravelli” Research Centre for Neurotechnology and Experimental Brain Therapeutics; 3 Department of Psychiatry, ASST Santi Paolo e Carlo; 4 Department of Psychology, University of Milano-Bicoccca; 5 ASST Santi Paolo e Carlo, Centro Giovani “Ettore Ponti”, Milan; 6 Clinical and Experimental Medicine , University of Pisa, Pisa, Italy

## Abstract

**Introduction:**

Nearly two-thirds of individuals with a mental disorder start experiencing symptoms during adolescence or early adulthood, and the onset of a mental disorder during this critical life stage strongly predicts adverse socioeconomic and health outcomes. Subthreshold manifestations of Autism Spectrum Disorders (ASD), also called autistic traits, are known to be associated with a higher vulnerability to the development of other psychiatric disorders. Current psychopathological research has pointed out the shortcomings of a categorical approach to mental illnesses (e.g., the concept of comorbidity) whilst a dimensional approach allows the description of patients across multiple syndrome dimensions that, in turn, constitute broad spectra of interrelated psychopathologies.

**Objectives:**

This study aimed to assess the presence of autistic traits in a population of young adults seeking specialist assistance, and to evaluate the study population across various psychopathological domains in order to determine their links with autistic traits.

**Methods:**

We recruited a sample of 263 adolescents and young adults referring to a specialized outpatient clinic, and we administered them several self-report questionnaires for the evaluation of various psychopathological domains: the *Autism Quotient,* the *Ritvo Autism and Asperger Diagnostic Scale-Revised *, RAADS-R, the *Empathy Quotient,* EQ, the *Sensory Perception Quotient – Short Form,* SPQ-SF35, the *Beck Depression Inventory*, BDI-II, the *State-Trait Anxiety Inventory,* STAI-Y1 and Y2, the *Eating Attitude Test-26 items*, EAT-26, the *Prodromal Questionnaire–short version*, PQ-16, the *Personality Inventory for DSM-5*, PID-5-BF, the *Pathological Narcissism Inventory*, PNI. We then conducted a cluster analysis based on the prevalence of autistic traits (AQ, RAADS-R), empathy (EQ) and sensory sensitivity (SPQ-SF35) scores.

**Results:**

The cluster analysis identified three distinct groups in the sample: an autistic traits (AT) cluster (22.43%), an intermediate cluster (45.25%), and a no-AT cluster (32.32%). Moreover, subjects with higher autistic traits exhibited significantly greater symptomatology across multiple psychopathological domains, including mood, anxiety, eating disorders severity, psychotic symptoms, and personality traits such as Detachment and Vulnerable Narcissism.

**Conclusions:**

The study highlights the importance of identifying autistic traits in young individuals struggling with mental health concerns, and suggests a relationship between autistic traits and greater overall psychopathological burden. Additionally, the findings underscore the necessity of adopting a dimensional approach to psychopathology, in order to better understand the complex interplay of different psychiatric symptoms and facilitate tailored interventions.

**Disclosure of Interest:**

None Declared

